# Clustering of methamphetamine users based on personality characteristics and self-efficacy in the west of Iran

**DOI:** 10.1038/s41598-024-66673-y

**Published:** 2024-07-09

**Authors:** Touraj Ahmadi Jouybari, Ali Zakiei, Safora Salemi, Zahra Lak, Mahsa Mohebian, João Maurício Castaldelli-Maia, Hafez Bajoghli, Sara Hookari, Mehran Kamani

**Affiliations:** 1 Substance Abuse Prevention Research Center, Health Institute, Kermanshah University of Medical Sciences, Kermanshah, Iran; 2 Sleep Disorders Research Center, Kermanshah University of Medical Sciences, Kermanshah, Iran; 3 Science and Research Branch, Faculty of Psychology, Islamic Azad University, Tehran, Iran; 4 Department of Neuroscience, Medical School, FMABC University Center, Santo André, SP Brazil; 5 Department of Psychiatry, Medical School, University of São Paulo, São Paulo, SP Brazil; 6 Iranian National Center for Addiction Studies (INCAS), Tehran University of Medical Sciences, Tehran, Iran; 7 Clinical Research Development Center, Imam Khomeini and Mohammad Kermanshahi and Farabi Hospitals, Kermanshah University of Medical Sciences, Kermanshah, Iran; 8 Sirjan School of Medical Sciences, Sirjan, Iran

**Keywords:** Methamphetamine, Personality traits, Self-efficacy, Mental health, Sleep quality, Relapse, Psychology, Medical research, Risk factors

## Abstract

With the substantial increase in the use of stimulants, especially methamphetamine, in recent years, the present study aimed to cluster methamphetamine users based on personality traits and self-efficacy, and compare their mental health, sleep quality, and the risk of relapse in the identified clusters. This cross-sectional study was conducted through convenience sampling on 501 methamphetamine users in addiction treatment centers in Kermanshah, western Iran. The data were collected using the Schwarzer General Self-Efficacy Scale, Zuckerman–Kuhlman Personality Questionnaire, Goldberg and Hiller General Health Questionnaire (GHQ), Zuckerman–Kuhlman Personality Questionnaire, and Stimulant Relapse Risk Scale (SRRS). A total of 501 methamphetamine users were distinguished into three clusters with frequencies of 111 (22.2%), 298 (59.5%), and 92 (18.4%) members through hierarchical cluster analysis. The participants in the first cluster were characterized by low self-efficacy, high neuroticism, sensation seeking, and aggressiveness, along with low extroversion and activity, low positive health, high negative health, low sleep quality, and high risk of drug relapse. The participants in the second cluster reported moderate levels of self-efficacy, neuroticism, sensation seeking, activity, and aggressiveness, high extroversion, and moderate levels of mental health, sleep quality, and the risk of relapse. Moreover, the participants in the third cluster reported the highest level of self-efficacy, the lowest level of neuroticism, sensation seeking, and aggressiveness, moderate extroversion and high activity, low relapse risk, high sleep quality, as well as high positive and low negative health symptoms. The third cluster was significantly different from the other two clusters in terms of the mentioned factors. The findings of this study suggest that low self-efficacy and the presence of neuroticism, sensation seeking, and high aggressiveness contribute to reduced mental health and sleep quality, as well as an increased risk of relapse in methamphetamine users.

## Introduction

In the past decades, the use of methamphetamine has increased rapidly around the world. For example, from 2009 to 2019, the amounts of methamphetamine detected in East and Southeast Asia increased almost tenfold. Chronic use of methamphetamine is associated with medical, psychiatric, and cognitive impairments^1,2^.

During 2015 and 2018, the estimated rate of past-year methamphetamine users among adults was 6.6 per 1000 individuals. Further, among those, about 27.3% reported to methamphetamine use for more than 200 days per year; 52.9% of those had a methamphetamine use disorder, and 22.3% injected methamphetamine^3^. In Iran, it is estimated that 1.6 to 2.67% of adult Iranians are addicted to drugs, in general, and after opium, amphetamine and methamphetamine are the most abused illicit substances^4^, in specific.

One of the side effects of methamphetamine use is decreased levels of mental health in users^5^. The use of methamphetamine has a profound negative effect on mental health, creates significant risks for mental health, and can also complicate the drug treatment procedure^6^. The burden of mental health and substance use disorders worldwide is predicted to increase in the coming decades^7^.

Previous studies have shown that people who use drugs have poor mental health, and mental disorders are often associated with the later onset of substance use disorders in these people^8–10^. Studies on the relationship between mental illness and substance use disorder have yielded mixed findings. Still, they generally reported common genetic, environmental, social, and cultural risk factors for poor mental health and substance use^11^. For example, cannabis use is associated with the risk of mental illness^12^. Conversely, mental illness may also increase the risk of substance abuse. People may use alcohol, tobacco, or amphetamine as self-medication to deal with distress and negative thoughts^13–15^.

In addition to the mentioned problems, long-term use of methamphetamine leads to harmful effects such as sleep problems in users^16^. Sleep is a natural body function that plays a crucial role in maintaining mental and physical health. Decreased sleep quality, increased sleep delay, and daytime sleepiness are often associated with methamphetamine use^17^. Moreover, symptoms such as neuroticism, depression, and decreased functioning associated with methamphetamine use can often lead to poor sleep quality^18^. Studies have shown that the consumption of methamphetamine can have many direct and indirect effects on the quality of sleep due to the disruption of the sleep–wake cycle^19,20^. The use of methamphetamine adversely affects the objective measures of sleep, increases the onset of sleep, and reduces the total sleep time significantly^20^.

Furthermore, relapse is one of the important complications of addiction, which occurs again after cessation of use. Relapse is the process of returning to past unhealthy behavior that forces and encourages a person to use the substance again. Substance use relapse is one of the main problems in treating people with drug addiction. For example, Hser et al.^21^ showed that 10–15% of methamphetamine users reported relapses 12 months after treatment. Brecht and Herbeck^22^ also found that 61% of methamphetamine users returned to methamphetamine use within 1 year after treatment and 25% within 2 to 5 years.

Addiction can be considered a physical, mental, and social disease, and many pre-addictive factors play an essential role in its formation and development^23^. Identifying these factors can be effective in controlling and preventing addiction. Self-efficacy is one of the variables associated with substance abuse^24^. Self-efficacy refers to people’s beliefs about their abilities to organize motivations and cognitive resources and control a given event. One of the fundamental aspects of self-efficacy is the belief that a person can influence the consequences of their life by exercising control, especially when facing stressors; having control over conditions is an important factor in adapting to various situations^23^.

Studies addressing substance use and self-efficacy have reported that self-efficacy is associated with people’s functioning. In other words, those who consider themselves more efficient and achieve functional success feel more satisfied with life and are less likely to engage in risky behaviors^25^. Dolan et al.^26^ showed that poor self-efficacy is the third reason for substance abuse in students. McKellar et al.^27^ examined issues related to alcohol consumption, depression, impulsivity, avoidant coping, and social support from friends. They found that self-efficacy is one of the main predictors among substance users. Robinson et al.^28^ showed that adolescents who have continuously avoided addictive substances were more efficient than others. Epstein et al.^29^ also showed that self-efficacy and problem-solving skills predict a high level of assertive refusal, leading to less substance use and smoking in a 2-year follow-up.

A literature review indicated that in addition to self-efficacy, personality traits are among the important cognitive factors in the tendency to abuse methamphetamine^30^. Indeed, from a psychological perspective, substance abusers have vulnerable personality traits^31^ that lead them to addiction. Personality traits refer to an organized set and a unit consisting of relatively fixed and stable characteristics in people, which distinguish a person from other people as a whole. Personality traits include countless sets of features whose impact on the body and mind of people is not the same. Some personality traits are closely related to their physical and mental health and well-being and have a decisive role and impact on their health^32,33^. In addition, some personality characteristics negatively correlate with neuroticism and mental problems^32^. Concerning the relationship between personality traits and addiction, Tsai et al. showed that lower levels of harm avoidance traits and higher levels of loneliness were significantly and positively associated with cravings in methamphetamine users during 1 year^30^. Another study found that people with high neuroticism and low conscientiousness are likelier to use drugs^34^.

Following these findings, investigating the factors associated with methamphetamine abuse is vital in the sense that methamphetamine abuse causes various problems in the health and well-being of users, such as increasing negative mental health, sleep problems, and the likelihood of relapse. Due to the high prevalence of methamphetamine use and the severe side effects this drug causes^4,35^, no study has addressed the problems of methamphetamine abusers through cluster analysis. It should be noted that cluster analysis is used to identify homogeneous clusters. In cluster analysis, the members in the same group (called a cluster) are more similar (in some sense) to each other than those in different clusters. Thus, cluster analysis is an exploratory tool that can reveal the concordance and structure of the data to understand the effective factors in methamphetamine abuse and provide solutions for prevention, treatment, and improving the health of methamphetamine abusers. Moreover, given the importance of methamphetamine use, the present study follows two goals: (1) To cluster methamphetamine users based on personality traits and self-efficacy and (2) To compare mental health, sleep quality, and the risk of relapse in the identified clusters.

## Methods

### Procedures and participants

The present study was a cross-sectional one. The study population included all methamphetamine users who visited the addiction treatment clinic of Farabi Hospital and the addiction treatment clinics (Kermanshah University of Medical Sciences, Kermanshah, Iran) and had been diagnosed with substance use disorder based on structured interviews (DSM-5) by a psychiatrist. The study’s enrollment criteria were using methamphetamine for at least 6 months, reading and writing literacy, and age over 18 years. The exclusion criteria were severe psychological problems (psychological records and mental disorders diagnosed by a psychologist and with the help of mental assessments), serious cognitive problems according to the psychiatrist’s diagnosis, ad any serious illness determined according to the doctor’s opinion and the patient’s medical records. The final sample size was estimated as 501 persons selected using convenience sampling from the study population who met the inclusion criteria during the study's timeframe. When determining the sample size, to ensure an adequate level of test power, a minimum of 20 samples per variable is considered sufficient^36^. Given that 22 sub-categories and overall scores are accounted for in this study, the minimum sample size required is 440. This study included 501 participants. Furthermore, hierarchical cluster analysis is deemed appropriate for datasets with fewer than 1000 cases, which applies to our study as well.

After obtaining permission from the Kermanshah University of Medical Sciences and making the necessary arrangements with the relevant authorities, the researchers visited the addiction treatment clinics and the applicants. They provided some information about the objectives of the study and obtained written and informed consent from the applicants. The eligible volunteers were evaluated through interviews by an experienced psychologist collaborating on the project. All participants were evaluated by completing the demographic checklist and other questionnaires regarding demographic information, individual and family self-report records, substance use records, and the data related to personality traits, self-efficacy, mental health, sleep quality, and risk of relapse. Data collection was done for almost 12 months. Questionnaires were completed from June, 2021–June 2022.

It should be noted that these data were collected through interviews with the patients by an experienced psychologist. The people with incomplete data for any reason were excluded from the study. Besides, all participants attended the study with full awareness of the details of the research project and its execution procedure. The participants’ data were collected anonymously, and the researcher ensured the participants that their data would remain with her confidentially. The questionnaires were administered at separate intervals to prevent the participants’ fatigue and non-cooperation. The interviews were conducted, and the questionnaires were completed after establishing rapport with the participants and considering their physical and mental conditions.

### Measures

#### Schwarzer general self-efficacy scale

The General Self-Efficacy scale has 10 items answered on a four-point Likert scale ranging from 1 to 4. In Schwarzer and Jerusalem’s study, Cronbach’s alpha coefficient for the whole scale ranged from 0.76 to 0.90, and the concurrent validity coefficient estimated for the scale was significantly correlated with emotions, optimism, and job satisfaction^37^. In Iran, in the research of Rabbani Bawjdan et al.^38^ the reliability of this scale using Cronbach’s alpha method with 354 samples is 0.82 Obtained.

#### Zuckerman–Kuhlman personality questionnaire

The Zuckerman–Kuhlman personality questionnaire is a self-report questionnaire used to measure 5 personality traits. Each item marked true is scored 1, and each false item is scored 0^39^. This study used the 41-item Persian version with established validity and reliability in Iran. The items cover neuroticism (10 items), sensation seeking (8 items), activity (8 items), extroversion (6 items), and aggressiveness (9 items)^40^.

#### General health questionnaire (GHQ)

The questionnaire was developed in 1972 by Goldberg and Hiller (1979) to identify mental disorders in different centers and environments. Its items examine the mental state in the last month and measure symptoms such as abnormal thoughts and feelings and aspects of observable behavior. Sánchez-López and Dresch^41^ examined the reliability and validity of the questionnaire. This study analyzed the internal consistency and external validity of the 12-item mental health questionnaire in the Spanish general population, including a stratified sample of 1001 individuals aged 25 to 65. In this study, Cronbach’s alpha for the questionnaire was estimated as 0.76. Within the Iranian context, the research undertaken by Ebadi et al., revealed that the internal consistency of the employed questionnaire, as measured by Cronbach's alpha, was found to be 0.87^42^.

#### The Pittsburgh sleep quality index (PSQI)

This self-report instrument was developed by Buysse (1989). It contains 18 items with a total score ranging from 0 to 21, with higher scores indicating lower sleep quality. Scores higher than 5 confirm poor sleep quality. In Iran, the Cronbach’s alpha coefficient for the PSQI was estimated as equal to 0.83, confirming the reliability of the instrument. In the current study, Cronbach’s alpha coefficient for the instrument was estimated as 0.91^43^.

#### Stimulant relapse risk scale (SRRS)

The scale assesses the risk of substance use disorder (SUD) relapse. This scale contains 30 items that measure 5 subscales, including anxiety and intention to use drugs (AI); emotionality problem (EP); compulsivity for drugs (CD); positive expectancies and lack of control over drugs (PL); and lack of negative expectancy for drugs (NE). The SRRS uses five items to assess insight into mental condition: awareness of illness (AI). These items are stated as follows: “The feeling I used to have while using the drug sometimes comes back” (AI); “I feel a constant need to put something in my mouth” (EP); “I would do almost anything to use the drug” (CD); “I would use the drug if I were alone” (PL); and “I would not be able to control myself if I use the drug” (NE). Higher average scores on the whole scale and subscale indicate a higher risk of relapse risk. In this study, only the total score was used. All items are related to a drug-related situation in the past 1 week. With 5 supplementary items measuring the respondent’s intensity of awareness of their illness, this scale contains 35 items. The items are assessed on a three-step rating scale, with higher scores indicating a greater risk of using the drug of dependence. Cronbach’s α coefficient for the 35-item SRRS in this study was 0.883, confirming its internal consistency^44^.

In the research conducted by Yamini and Khorsandi Shamir^45^ in Iran, the construct validity of the questionnaire, encompassing both convergent and divergent aspects, was found to be at a commendable level. Furthermore, the Cronbach’s alpha coefficients, a measure of internal consistency, were calculated to be 0.94 for the instrument as a whole, and ranged 0.95 for its constituent sub-scales.

### Statistical analysis

The recorded data were entered into SPSS-24 software by a statistician and analyzed using relevant statistical techniques. The personality traits, self-efficacy, mental health, sleep quality, and risk of relapse in methamphetamine users were assessed with the help of cluster analysis. The k-nearest neighbor’s algorithm and the squared Euclidean distance were used in the hierarchical cluster analysis. The final number of clusters was specified using cluster analysis and drawing a dendrogram and cumulative values. Descriptive statistics were used to evaluate clusters to determine the distribution of demographic factors in frequency, percentage, minimum, maximum, mean, and standard deviation. The chi-square test was used to check the relationship between the distribution of classified factors in the studied clusters. If necessary, the Fisher exact test was used to adjust it. The average values for the quantitative factors in the extracted clusters were compared through a one-way analysis of variance (ANOVA). Ultimately, the data from 501 participants were entered into the software and analyzed using the relevant statistical methods. The significance level was set at 0.05.

### Ethical considerations

This study was conducted with full compliance with ethical protocols. To this end, the objectives of the study were explained to the participants in clear and simple language. Participation in the study was completely voluntary and without any compulsion, and the participants were assured that the results of the analysis and tests would be kept completely confidential. Moreover, the participants’ identity information was not recorded, and only the codes reported by the participants were used to identify the data. The participants had the right to withdraw at any study stage, including data collection. Written informed consent was obtained from all participants who signed a consent form. This study was registered at Kermanshah University of Medical Sciences in Iran and was approved by the university's ethics committee (IR.KUMS.REC.1399.1179.).

## Results

The data for 501 methamphetamine users were analyzed using hierarchical cluster analysis. The k-nearest neighbors algorithm was used in the hierarchical cluster analysis to determine the number of clusters based on the self-efficacy and personality traits of the participants. This algorithm calculates the distance between two clusters based on the distance between their farthest points. Besides, the squared Euclidean distance was considered a measure of cluster similarity. The data analysis, the dendrogram, and the accumulation coefficients and reducing their changes along with the increase in the number of clusters showed that participants were placed into three clusters according to the level of self-efficacy and personality traits. Accordingly, different self-efficacy and personality trait averages created three clusters in the studied sample. In the end, the participants were distinguished into three clusters with frequencies of 111 (22.2%), 298 (59.5%), and 92 persons (18.4%), respectively (Table 1).

**Table 1:** Cluster analysis with the k-nearest neighbors algorithm.

Clusters (n = 501)			Accumulation coefficients			
Frequency (%)						
Cluster 1	Cluster 2	Cluster 3	Changes in coefficients	Last-stage coefficients	Last-stage accumulation coefficients	The maximum possible number of clusters
111 (22.2%)	298 (59.5%)	92 (18.4%)	487.00	552.00	1039.00	2
			213.00	339.00	552.00	3
			46.00	293.00	339.00	4

An analysis of the demographic characteristics, personal-family histories, and substance use data of the three clusters indicated that the participants in the clusters were significantly different in terms of their current age and gender distribution (all Ps < 0.05). Although none of the participants in the third cluster were over 55 years old, the number of participants age group in the first cluster (7 persons; 6.3%) was three times greater than that of the participants in the second cluster (5 persons 1.7%). Moreover, the participants aged 15–35 accounted for more than half of the participants in the first cluster (58 persons; 52.3%) and the third cluster (48 persons; 52.7%). However, the participants aged 36–55 in the second cluster accounted for more than half of the participants (151 persons; 50.7%). Thus, the second cluster with a higher current age distribution than the average age distribution in the first cluster and low age distribution in the third cluster showed that cluster memberships are significantly different based on the current age of the participants. Moreover, the number of women in the third cluster (17 persons; 18.5%) was reported to be more than five times of the women in the second cluster (10 persons; 3.4%) and about one and a half times of the women in the first cluster (14 persons; 12.6%), showing significant gender differences and distinctions in terms of the number of male participants in the first and third clusters and the female participants in the second cluster.

The clusters defined in terms of the history of legal problems in the family as well as the history of physical and psychological abuse in childhood, showed statistically significant differences. Thus, the history of family legal problems was reported in the first cluster (23 cases; 20.9%) more than one and a half times the second cluster (38 cases; 12.6%) and more than twice the third cluster (8 cases; 8.7%). Moreover, in the first cluster, the number of mental abuse (15 cases; 13.5%) and physical abuse cases (29 cases; 26.1%) in childhood was significantly higher compared to the second and third clusters. An analysis of the distribution of a history of substance use cases among the three clusters showed that the clusters had statistically significant differences in substance use duration, family history of substance use, and the first reason for substance use (all Ps < 0.05). The period of substance use was more than 72 months in the first cluster with (79 cases; 70.8%), nearly one and a half times the second cluster (150 cases; 50.5%), and more than one and a half times the third cluster with 41 cases (44.0%). The analysis of the reasons for the first substance use also showed that most participants in the first cluster reported peer persuasion as the first reason for their substance use (115 persons; 38.6%) and seeking pleasure (34 persons; 30.6%). At the same time, most participants in the second and third clusters reported getting pleasure and persuasion by friends as the first reason for their substance use. Family problems reported as the first reason for substance use in the third cluster (14 persons; 15.4%) were significantly higher than the first cluster (5 cases; 4.5%) and the second cluster (13 cases; 4.4%) (Table 2).

**Table 2:** The descriptive statistics for the participants’ demographic characteristics and their self-report conditions.

	Variable	Categories	Clusters			Total (n = 501)	Chi-square (P-value)
			Cluster 1 (n = 111)	Cluster 2 (n = 298)	Cluster 3 (n = 92)		
Demographic characteristics	Patient current age	15–35	58 (52.3)	142 (47.7)	48 (52.7)	248 (49.6)	11.90* (0.018)
		36–55	46 (41.4)	151 (50.7)	43 (47.3)	240 (48.0)	
		56–75	7 (6.3)	5 (1.7)	0 (0.0)	12 (2.4)	
	Gender	Female	14 (12.6)	10 (3.4)	17 (18.5)	41 (8.2)	25.12*** (< 0.0001)
		Male	97 (87.4)	288 (96.66)	75 (81.5)	460 (91.8)	
	Education	Lower education	64 (57.7)	191 (64.1)	50 (54.3)	305 (60.9)	3.44 (0.488)
		Diploma and associate degree	40 (36.0)	91 (30.5)	36 (39.1)	167 (33.3)	
		Graduate and postgraduate degrees	7 (6.3)	16 (5.4)	6 (6.5)	29 (5.8)	
	Occupation	Unemployed	50 (45.5)	101 (33.9)	35 (38.0)	186 (37.1)	4.350 (0.114)
		Self-employed	61 (55.0)	197 (66.1)	57 (620)	315 (62.9)	
	Marital status	Single	56 (50.5)	180 (60.4)	46 (50.0)	282 (56.3)	11.81 (0.066)
		Married	37 (33.3)	98 (32.9)	34 (37.0)	169 (33.7)	
		Divorced	13 (11.7)	16 (5.4)	10 (10.9)	39 (7.8)	
		Widow	5 (4.5)	4 (1.3)	2 (2.2)	11 (2.2)	
Individual and family history	Previous divorce	Yes	18 (16.2)	33 (11.1)	15 (16.3)	66 (13.2)	2.83 (0.242)
		No	93 (83.8)	265 (88.9)	77 (83.7)	435 (86.8)	
	Addiction-related divorce	Yes	10 (12.2)	28 (12.8)	9 (12.9)	47 (12.7)	0.022 (0.989)
		No	72 (87.8)	191 (87.2)	61 (87.1)	324 (87.3)	
	Housing	Parental house	42 (54.5)	136 (65.7)	40 (58.8)	218 (61.9)	5.144 (0.525)
		Rental house	21 (27.3)	45 (21.7)	15 (22.1)	81 (23.0)	
		Personal house	12 (15.6)	21 (10.1)	12 (17.6)	45 (12.8)	
		Living in a camp	2 (2.6)	5 (2.4)	1 (1.5)	8 (2.3)	
	Economic status	Very poor	5 (4.5)	19 (6.4)	6 (6.6)	30 (6.0)	10.69 (0.098)
		Poor	36 (32.4)	105 (35.6)	30 (33.0)	172 (34.3)	
		Moderate	51 (45.6)	148 (49.77)	38 (41.8)	237 (47.3)	
		Good	19 (17.1)	25 (8.4)	17 (18.7)	61 (12.2)	
		Very good	0 (0.0)	0 (0.0)	0 (0.0)	0 (0.0)	
	Legal convictions	Yes	33 (29.7)	87 (29.2)	23 (25.3)	143 (28.6)	0.614 (0.736)
		No	78 (70.3)	211 (70.8)	68 (74.7)	357 (71.4)	
	Physical illness	Yes	16 (14.4)	36 (12.1)	10 (10.9)	62 (12.4)	0.642 (0.725)
		No	95 (85.6)	262 (87.9)	82 (89.1)	439 (87.6)	
	Mental illness	Yes	16 (14.4)	32 (10.7)	7 (7.66)	55 (11.0)	2.427 (0.297)
		No	95 (85.6)	266 (89.3)	85 (92.4)	446 (89.0)	
	Physical illness in the family	Yes	16 (14.4)	31 (10.4)	15 (16.5)	62 (12.4)	2.91 (0.234)
		No	95 (85.6)	267 (89.6)	76 (83.5)	439 (87.6)	
	Mental illness in the family	Yes	6 (7.1)	22 (10.0)	5 (7.4)	33 (8.8)	0.863 (0.649)
		No	79 (92.9)	199 (90.0)	63 (92.6)	341 (91.2)	
	Legal convictions in the family	Yes	23 (20.9)	38 (12.6)	8 (8.7)	59 (11.8)	6.7707** (0.007)
		No	88 (79.1)	260 (87.4)	84 (91.3)	441 (88.2)	
	Mental harassment in childhood	Yes	15 (13.5)	15 (5.1)	8 (8.8)	38 (7.6)	8.44* (0.015)
		No	96 (86.5)	282 (94.9)	83 (91.2)	461 (92.4)	
	Physical harassment in childhood	Yes	29 (26.1)	28 (9.4)	8 (8.8)	65 (13.0)	21.76*** (< 0.0001)
		No	82 (73.9)	270 (90.6)	83 (91.2)	435 (87.0)	
Substance use history	The age at the onset of substance use	10–19	45 (40.5)	101 (33.9)	29 (31.5)	175 (34.9)	12.52 (0.051)
		20–29	41 (36.9)	141 (47.3)	36 (39.1)	218 (43.5)	
		30–39	20 (18.0)	48 (16.1)	18 (19.6)	86 (17.2)	
		> 39	5 (4.5)	8 (2.7)	9 (9.8)	22 (4.4)	
	Substance use (duration)	0–24	12 (11.1)	67 (22.5)	18 (20.0)	76 (15.2)	23.91*** (< 0.0001)
		25–48	10 (9.1)	30 (9.9)	18 (20.0)	51 (10.2)	
		49–72	10 (9.1)	51 (17.1)	15 (16.0)	59 (11.8)	
		> 72	79 (70.8)	150 (50.5)	41 (44.0)	315 (62.9)	
	Alcohol consumption in the family	Yes	38 (34.2)	69 (23.2)	33 (53.9)	140 (27.9)	8.44** (0.015)
		No	73 (65.8)	229 (76.8)	59 (64.1)	361 (72.1)	
	Alcohol consumption	Yes	6 (5.4)	17 (5.7)	3 (3.3)	26 (5.2)	0.867 (0.648)
		No	105 (94.6)	281 (94.3)	89 (96.7)	475 (94.8)	
	The reason for the first substance use	Pleasure	34 (30.6)	115 (38.6)	22 (24.2)	171 (34.2)	30.24** (0.001)
		Peer persuasion	39 (35.1)	86 (28.9)	21 (23.1)	146 (63.4)	
		Curiosity	17 (15.3)	46 (15.4)	21 (23.1)	84 (16.8)	
		Alleviating physical pain	3 (2.7)	20 (6.7)	7 (7.7)	30 (6.0)	
		Family problems	5 (4.5)	13 (4.4)	14 (15.4)	32 (6.4)	
		Alleviating mental problems	13 (2.6)	18 (3.6)	6 (1.2)	37 (7.4)	

*Significant at P < 0.05.

**Significant at P < 0.01.

***Significant at P < 0.001.

A comparison of the participants’ average current age, the age of the first substance use, and the duration of substance use in the three clusters revealed that the mentioned variables showed statistically significant differences among the three groups (P < 0.05). Accordingly, the average age of the first substance use for the participants in the third cluster (25.48) was significantly higher than that of the first cluster (22.74) and the second cluster (23.49). Besides, the average duration of substance use for the participants in the third cluster (109.17) was significantly lower compared to the first cluster (146.2) and the second cluster (121.4) (Table 3).

**Table 3:** Comparing the average current age, the first substance use, and duration of substance use in the three clusters.

	Mean (std. deviation) [min–max]				F (P value)	Clusters compared	Mean difference (P-value)
	Cluster 1 (n = 111)	Cluster 2 (n = 298)	Cluster 3 (n = 92)	Total (n = 501)			
Participants’ data							
Current age	35.32 (10.97) [18–65]	36.55 (9.12) [16–65]	35.01 (8.64) [20–54]	23.46 (6.01)	1.371 (0.255)	(1), (2)	− 1.32 (0.420)
						(1), (3)	0.214 (0.986)
						(2), (3)	1.54 (0.362)
Age at the onset of substance use	22.74 (6.91) [11–58]	23.49 (8.72) [12–60]	25.48 (11.11) [12–60]	23.41 (8.34) [11–60]	3.845 (0.022)*	(1), (2)	− 0.784 (0.696)
						(1), (3)	− 2.74 (0.016)*
						(2), (3)	− 1.99 (0.205)
Substance use duration (month)	146.2 (101.6) [1–492]	1214.1 (115.11) [1–540]	109.1 (93.75) [2–420]	133.95 (104.35) [1–540]	5.554 (0.004)**	(1), (2)	24.78 (0.080)
						(1), (3)	− 37.07 (0.008)***
						(2), (3)	12.31 (0.676)

*Significant at P < 0.05.

**Significant at P < 0.01.

**Significant at P < 0.001.

Further analysis indicated that the average self-efficacy score for the participants in the third cluster (32.41) was significantly higher than the score of the participants in the second cluster (23.86) and the first cluster (15.76). Moreover, a comparison of the average scores of personality traits among the three clusters indicated that the average scores for neuroticism, sensation seeking, extroversion, and aggressiveness showed statistically significant differences among the clusters (P < 0.05). Hence, the average scores for neuroticism, excitement, and aggressiveness were reported to be higher in the first cluster compared to the second cluster, and the corresponding values were higher in the second cluster than in the third cluster. However, the mean score for extraversion was higher in the second cluster compared to the first and third clusters, and the corresponding value was higher in the third cluster compared to the second cluster. This means that higher levels of extraversion are associated with higher levels of self-efficacy. The data analysis also indicated self-efficacy, and each of the subscales of neuroticism, sensation seeking, extroversion, and aggressiveness played a statistically significant role in differentiating the determined clusters and assigning methamphetamine users to these clusters (all Ps < 0.05). These results also showed that participants in the first cluster were characterized by high neuroticism, sensation seeking, aggressiveness, low extroversion, activity, and self-efficacy. The participants in the second cluster reported moderate self-efficacy and neuroticism, sensation seeking, moderate activity and aggressiveness, and high extroversion. Furthermore, the participants in the third cluster were distinguished from the members of the other two clusters with the highest level of self-efficacy, low levels of neuroticism, sensation seeking, and aggressiveness, moderate extroversion, and high activity (Table 4).

**Table 4:** Comparing self-efficacy and personality traits in the three clusters.

	Mean (std. deviation) [min–max]				F (P-value)	Clusters compared	Mean difference (P-value)
	Cluster 1 (n = 111)	Cluster 2 (n = 298)	Cluster 3 (n = 92)	Total (n = 501)			
Self-efficacy	3.43 (15.76) [10–22]	2.26 (23.86) [18–29]	3.75 (32.41) [26–40]	6.01 (23.64) [10–40]	84.83 (< 0.0001)***	(1), (2)	− 8.10 (< 0.0001)***
						(1), (3)	− 16.64 (< 0.0001)***
						(2), (3)	− 8.55 (< 0.0001)***
Personality traits							
Neuroticism	2.32 (6.99) [1–10]	2.29 (5.11) [0–9]	2.87 (4.78) [0–10]	2.55 (5.47) [0–10]	28.82 (< 0.0001)***	(1), (2)	1.87(< 0.0001)***
						(1), (3)	2.20 (< 0.0001)***
						(2), (3)	0.33(0.479)
Sensation seeking	2.82 (5.27) [0–9]	2.34 (5.13) [0–9]	2.75 (4.33) [0–9]	2.54 (5.02) [0–9]	4.189 (0.016)*	(1), (2)	0.136(0.897)
						(1), (3)	0.933 (0.025)*
						(2), (3)	0.797 (0.023)*
Activity	2.13 (4.04) [0–7]	1.43 (4.26) [0–7]	1.43 (4.58) [1–7]	1.62 (4.27) [0–7]	2.84 (0.059)	(1), (2)	− 0.22 (0.446)
						(1), (3)	− 0.54 (0.047)*
						(2), (3)	− 0.323 (0.215)
Extroversion	1.93 (3.43) [0–7]	1.70 (4.17) [0–8]	1.96 (3.84) [0–8]	1.83 (3.95) [0–8]	7.055 (< 0.001)**	(1), (2)	− 0.75 (0.001)***
						(1), (3)	− 0.41 (0.234)
						(2), (3)	0.329 (0.278)
Aggressiveness	2.31 (6.22) [1–10]	2.11 (5.41) [0–10]	2.22 (5.13) [0–9]	1.83 (3.95) [0–8]	7.59 (< 0.001)**	(1), (2)	0.805 (0.003)***
						(1), (3)	1.09 (0.001)***
						(2), (3)	0.289 (0.505)

*Significant at P < 0.05.

**Significant at P < 0.01.

***Significant at P < 0.001.

Furthermore, a comparison of the average scores of positive health and negative health among the three clusters showed that the two components of health have a statistically significant effect in distinguishing the clusters from each other. Thus, the participants in the second and third clusters had higher levels of positive signs of mental health and lower levels of negative health. In addition, the average scores for positive health symptoms in each cluster showed statistically significant differences compared to the first cluster. The average negative health score also showed a statistically significant difference between the first and second clusters (Ps < 0.05). An analysis of the average scores for sleep quality and its sub-scales also showed that sleep efficiency and sleep disorders, as well as the overall score of sleep quality, played a significant role in differentiating the clusters from each other (Ps < 0.05). Thus, mean negative health, overall sleep quality, sleep disorders, and sleep efficiency were significantly higher in the first cluster compared to the second cluster, and the corresponding values in the second cluster were greater compared to the third cluster.

Furthermore, positive health was significantly higher in the third cluster compared to the second and first clusters. Further examination of the differences between the clusters showed that the risk of relapse of stimulant use and all its sub-scales also had a statistically significant effect on the formation and differentiation of the three clusters (Ps < 0.05). Following these results, the higher levels of self-efficacy in the second and third clusters were associated with lower levels of relapse risk symptoms in these two clusters compared to the first cluster.

On the other hand, the average scores of relapse risk and its sub-subscales and the average overall scores of sleep quality, sleep efficiency, and sleep disorders were significantly higher in the first cluster compared to the second and third clusters (Ps < 0.05). These findings indicated that the participants in the first cluster reported low self-efficacy, high relapse risk, low positive health symptoms, high negative health symptoms, and low sleep quality. Thus they were differentiated from the participants in the other two clusters. The second cluster was distinguished from the other clusters with a moderate level of self-efficacy, mental health, sleep quality, and the risk of relapse. On the other hand, the third cluster was characterized by high self-efficacy, low risk of relapse, and low sleep quality score, as well as high positive health and low negative health signs (Table 5).

**Table 5:** Comparing self-efficacy and personality traits in the three clusters.

	Mean (std. deviation) [min–max]				F (P-value)	Clusters compared	Mean difference (P-value)
	Cluster 1 (n = 111)	Cluster 2 (n = 298)	Cluster 3 (n = 92)	Total (n = 501)			
Mental health							
Positive health	5.64 (2.88) [0–14]	8.25 (3.07) [0–18]	8.88 (3.58) [0–18]	7.78 (3.34) [0–18]	34.916*** (< 0.0001)	(1), (2)	− 0.261*** (< 0.0001)***
						(1), (3)	− 3.24 (< 0.0001)***
						(2), (3)	− 0.63 (0.213)
Negative health	8.21 (3.65) [0–18]	7.07 (3.82) [0–16]	7.04 (5.23) [0–18]	7.32 (4.10) [0–18]	3.375* (0.035)	(1), (2)	1.134 (0.034)*
						(1), (3)	1.164 (0.108)
						(2), (3)	0.030 (0.998)
Sleep quality							
Subjective sleep quality	1.63 (1.08) [0–3]	1.60 (0.85) [0–3]	1.42 (0.97) [0–3]	1.58 (0.93) [0–3]	1.56 (0.212)	(1), (2)	0.026 (0.965)
						(1), (3)	0.208 (0.257)
						(2), (3)	0.180 (0.236)
Delayed sleep onset	1.49 (0.79) [0–3]	1.48 (0.69) [0–3]	1.41 (0.69) [0–3]	1.47 (0.72) [0–3]	0.404 (0.668)	(1), (2)	0.013 (0.986)
						(1), (3)	0.082 (0.693)
						(2), (3)	0.069 (0.694)
Sleep duration	1.32 (1.35) [0–3]	1.58 (1.21) [0–3]	1.39 (1.19) [0–3]	1.49 (1.24) [0–3]	2.12 (0.121)	(1), (2)	− 0.258 (0.147)
						(1), (3)	0.066 (0.922)
						(2), (3)	0.191 (0.398)
Sleep efficiency	0.99 (1.23) [0–3]	0.78 (1.18) [0–3]	0.68 (1.06) [0–3]	0.88 (1.19) [0–3]	3..205* (0.041)	(1), (2)	0.212 (0.291)
						(1), (3)	− 0.311 (0.050)*
						(2), (3)	− 0.097 (0.828)
Sleep disorders	1.83 (0.66) [0–3]	1.73 (1.02) [0–3]	1.56 (0.63) [0–3]	1.76 (0.76) [0–3]	4.459* (0.021)	(1), (2)	0.100 (0.455)
						(1), (3)	0.264 (0.009)**
						(2), (3)	0.164 (0.268)
Taking hypnotics	1.45 (1.18) [0–3]	1.36 (0.93) [0–3]	1.19 (1.05) [0–3]	1.35 (1.01) [0–3]	1.618 (0.199)	(1), (2)	0.092 (0.113)
						(1), (3)	0.245 (0.143)
						(2), (3)	0.161 (0.121)
Circadian rhythm disorder	1.38 (1.05) [0–3]	1.48 (0.79) [0–3]	1.32 (0.86) [0–3]	1.43 (0.87) [0–3]	1.455 (0.234)	(1), (2)	− 0.100 (0.097)
						(1), (3)	0.063 (0.123)
						(2), (3)	0.163 (0.104)
Total sleep quality	10.32 (4.00) [1–20]	9.69 (5.54) [0–21]	9.08 (4.13) [1–18]	9.96 (4.42) [0–21]	3.050* (0.048)	(1), (2)	0.635 (0.398)
						(1), (3)	1.242 (0.049)*
						(2), (3)	0.606 (0.593)
Substance use relapse							
Neuroticism and intention to use	23.52 (5.72) [10–36]	21.04 (4.90) [8–34]	19.80 (5.21) [10–32]	21.31 (5.2) [8–36]	12.19*** (< 0.0001)	(1), (2)	2.21 (< 0.0001)***
						(1), (3)	3.45 (< 0.0001)***
						(2), (3)	1.24 (0.110)
Emotional problems	28.60 (6.00) [16–45]	24.39 (4.84) [13–39]	23.93 (5.56) [12–36]	25.24 (5.5) [12–45]	29.51*** ((< 0.0001)	(1), (2)	4.21 (< 0.0001)***
						(1), (3)	4.68 (< 0.0001)***
						(2), (3)	0.467 (0.736)
Compulsion to use	10.98 (3.92) [4–20]	9.67 (3.17) [4–18]	9.64 (3.57) [4–18]	9.95 (3.46) [4–20]	6.389*** (< 0.002)	(1), (2)	1.31 (0.002)***
						(1), (3)	1.34 (0.016)*
						(2), (3)	0.030 (0.997)
Positive expectations and lack of control over substance use	15.26 (4.25) [6–25]	13.30 (3.50) [5–25]	13.23 (3.94) [5–23]	13.72 (3.8) [5–25]	11.59*** (< 0.0001)	(1), (2)	1.96 (< 0.0001)***
						(1), (3)	2.03 (< 0.0001)***
						(2), (3)	0.073 (0.986)
Lack of negative expectations from the substance	10.45 (3.22) [4–19]	11.69 (3.08) [4–20]	11.05 (3.44) [4–19]	11.30 (3.2) [4–20]	6.370*** (< 0.002)	(1), (2)	− 1.23 (0.002)
						(1), (3)	− 0.59 (0.382)
						(2), (3)	0.935 (0.217)
Total relapse risk	104.57 (16.43) [72–145]	95.20 (13.0) [62–134]	93.14 (15.6) [61–130]	96.89 (14.9) [61–145]	21.113** (< 0.0001)	(1), (2)	9.37 (< 0.0001)***
						(1), (3)	11.43 (< 0.0001)***
						(2), (3)	2.06 (0.452)

## Discussion

The present study compared self-efficacy and personality traits based on cluster analysis of mental health, sleep quality, and relapse among methamphetamine users. This study used self-efficacy and personality traits to cluster methamphetamine users. The results of the data analysis showed that the level of self-efficacy was lower in the first cluster than in the second and third clusters. Accordingly, negative mental health was higher, and positive mental health was lower in the same cluster. Furthermore, the substance users in this cluster had a lower quality of sleep and a higher relapse.

Our findings indicated that self-efficacy is associated with both negative and positive mental health outcomes. Previous research by Yıldırım and Güler^46^ and Schalk and Reynaert Gloudemans^47^ also supported the connection between self-efficacy and mental health. This suggests that self-efficacy plays a role in enhancing individuals’ health by promoting adaptive coping mechanisms and shielding them from psychological distress^48^.

The present study’s data revealed that methamphetamine users in the first cluster, characterized by low self-efficacy, reported lower sleep quality, which is consistent with previous studies (e.g., Schlarb et al.; Byun et al.; Ghodrati Mirkouhi et al.; Miró et al.; Tighe et al.; Simonetti et al.)^49–54^. This finding suggests that lifestyle, which can be influenced by an individual’s self-efficacy, plays a role in sleep quality. Self-efficacy, as a psychological ability, has the potential to alleviate sleep problems. Self-efficacy can regulate negative feelings and enhance emotional well-being, ultimately improving sleep quality^55^.

Methamphetamine users in the first of our clusters, characterized by lower self-efficacy, exhibited the highest relapse rate compared to the second and third clusters. This finding aligns with Zhang et al., who found that individuals with low self-efficacy had more frequent relapses and tended to perceive relapse as a personal failure^56^. Schuck et al. also demonstrated the effectiveness of increasing self-efficacy in preventing smoking relapse during stressful and tempting situations^57^. These findings support the notion that self-efficacy, as a personality trait, can influence substance use relapse. Self-efficacy, rooted in purposeful thinking, is crucial in individuals' behavior in high-risk situations. Those with high self-efficacy are better equipped to resist substance temptation, leading to a lower likelihood of relapse. For instance, participants in the third cluster, characterized by higher self-efficacy, exhibited lower relapse rates compared to those in the other two clusters^24,58,59^.

In addition to self-efficacy, personality traits were used in the present study to cluster methamphetamine users. The data showed that neuroticism was high in the cluster with low mental health and sleep quality and a high drug relapse rate. However, the participants with low neuroticism in the third cluster reported different results, indicating that mental health and sleep quality were high and relapse rates were low. Lee et al.^60^ confirmed the relationship between neuroticism and mental health. Shokrkon and Nicoladis^61^ also found that people who have a high neuroticism score usually experience more negative emotions such as neuroticism, anger, irritability, and fear and show a stronger reaction to stressors, making them vulnerable to the adverse consequences of stressful experiences and thus feeling more anxious and insecure^62^.

The findings of the present study also demonstrated that neuroticism is associated with sleep quality in methamphetamine users. For instance, sleep quality was lower in the participants in the first cluster with higher neuroticism compared to the second and third clusters. The impact of neuroticism on the quality of sleep and frequent awakenings during sleep has been confirmed by other studies in the literature^63–65^. People usually experience stress and mental suffering when faced with changes, and the changes made in their life may lead to poor sleep quality. On the other hand, considering that stress and neuroticism are formed through physiological-psychological arousal, such changes certainly affect the quality of sleep^66,67^.

In addition to sleep quality, neuroticism was also associated with the relapse rate in methamphetamine users. As a case in point, the participants in the first cluster, with higher neuroticism, reported a higher relapse score rate. Schellekens et al.^68^ also found that the relapse rate is related to neuroticism. In line with this finding, Fisher et al.^69^ found that people with high neuroticism have a higher relapse rate. Hojjat et al.^70^ also showed that high neuroticism is associated with more methamphetamine use. These people show a weaker degree of adaptability with others and psychological distress, and these characteristics in people with neurotic traits probably make them more prone to relapse^71^.

The present study showed that, compared to the other two clusters, the sensation-seeking score was high in methamphetamine users in the first cluster. They reported lower positive mental health symptoms, higher negative health symptoms, lower sleep quality, and a higher relapse rate. Kalantari and Zolfaghari Zaferani also found a correlation between sensation seeking and general health. Accordingly, we can argue that excitement-seeking people do various things to get the optimal arousal level, which can adversely affect their mental health^72^.

The data in this study indicated that in addition to mental health, sensation seeking was also associated with the quality of sleep in methamphetamine users, indicating that the higher the sensation seeking, the lower the sleep quality and vice versa, as reported in previous studies^73–75^. This is to argue that high sensation seeking can increase emotional response through cognitive hyperarousal and facilitate insomnia.

Sensation seeking was related to substance abuse relapse in the present study. Accordingly, in the third cluster, compared to the first cluster in which sensation seeking was low, the relapse rate was lower compared to the first and second clusters. Hampson, Andrews, and Barkley^76^ also found that sensation-seeking, bonding, and social perceptions are predictors of substance abuse. In another study, Martins et al.^77^ studied two clusters of people with severe substance dependence and normal people and concluded that sensation seeking and impulsivity have a positive relationship with substance use. This finding implies that negative emotions and the inability to manage them properly are important triggers for the resumption of substance use, and negative emotions make people vulnerable to addiction and its recurrence^78^.

Another variable that was addressed in cluster analysis was aggressiveness. The participants in the first cluster reported a higher level of aggressiveness compared to the second and third clusters. Besides, mental health and sleep quality were reported to be lower in the same cluster, and the relapse rate was higher. This finding suggests that aggressiveness is one of the important and inherent human emotions and is a common reaction to failure and misbehavior. This emotion has unquestionable importance in terms of evolution. Still, its indiscriminate and excessive use causes some health and interpersonal problems for people because quick judgment, faulty information processing, and incorrect predictions can pave the way for aggressive behaviors, leading to negative mental health in people using methamphetamine^79^.

The data in this study also indicated that aggressiveness was higher in people with sleep problems in the first cluster, as reported by Afshar and Banisi^66^. Accordingly, we can argue that aggressiveness is one of the complex human emotions and a common reaction to failure and misbehavior. Aggressiveness is a type of negative emotion. Expressing anger and aggressiveness during the day can cause sleep problems and lead to feeling tired and sleepy during the day. In general, more aggressive people fall asleep later and have poor sleep quality. Nevertheless, it should also be noted that sleep problems have a two-way relationship with aggressiveness. For example, Kayser et al.^80^ reported that sleep disorders negatively affect many functions and are related to aggressiveness and violence, as confirmed by Kamphuis et al.^80^.

The present study found that methamphetamine users in the first cluster with a high level of aggressiveness also reported a higher relapse rate. McCormick and Smith^81^ found that subjects who scored higher on aggressiveness and aggressiveness measures reported more situations that caused them to use substances and had less confidence that they would withstand such situations. This was especially true for situations involving unpleasant moods, rejection, and conflict with family and friends. Likewise, Plüddemann et al.^82^ found that individuals who used methamphetamine engaged significantly more frequently in aggressive behaviors. These findings imply that problems caused by impulsivity, such as weak inhibition, decision-making, and planning, can be major obstacles in treating people with substance use disorders, especially in initiating, following up, and continuing treatment. In clinical samples, impulsivity is associated with factors that play a role in relapse, such as temptation and severity of substance use, and impulsivity is a potential mediator in treatment response and effectiveness. Impulsive behaviors can lead to the formation of a strong desire to use in substance users, and as a result, undermine their inability to resist it and contribute to returning to substance use again^83^.

Another construct of personality traits that were addressed in this study is extroversion. The findings indicated that extroversion was low in the participants with lower levels of mental health and sleep quality in the first cluster. This finding was consistent with the results reported by Umegaki and Higuchi^84^ and in contrast with the observations made by Klinger-König et al.^85^. They reported that low extroversion could predict high mental health. Accordingly, it can be argued that extroversion is partly related to individual differences. That is, extroverted people show relatively high levels of happiness, enthusiasm, energy, interest, and tirelessness. Studies have also demonstrated that extroverted people are highly motivated to interact with others and spend more time in the socialization process, and use of social support^86^. Such people also benefit from social support, socializing, and warm and intimate relationships with others. Hence, extroverted people are less passive and self-blaming and are less likely to retreat to isolation, and they experience higher mental health. In contrast, low extroversion is likely to have a positive effect on mental health^87^.

In a study on 22,000 adults aged 30 to 107 years, Stephan et al.^88^ found that higher scores in extraversion were associated with better sleep quality. This finding was also confirmed by Hintsanen et al.^89^ and Gray and Watson^90^. Extroverted people have better mental and physical health and are less reactive to stressful factors, which may lead to better sleep quality^91^. Since extraversion is associated with less reactivity to stress, lower risk of lung disease, better respiratory function, and a more physically active lifestyle, extrovert people are less likely to suffer from sleep problems^88^.

Accordingly, the findings of the present study confirmed a lower relapse rate in people with high extroversion. Other studies have also reported that high extraversion is the most important predictor for starting and increasing the consumption of drugs such as tobacco, alcohol, and cigarettes^48,92^. This is to argue that extroversion is considered a basic personality trait and is characterized by energy, dominance, positive emotions, and sociability, and it is possible that when this personality trait is lower in people, the rate of relapse will increase^93^.

### Limitation

The present study was conducted with some limitations. For instance, the participants were methamphetamine users in Kermanshah and were selected through convenience sampling. Thus, the findings might have less generalizability to other populations. A quantitative method was used for data collection. Thus, future studies need to conduct semi-structured interviews to collect more data. Future researchers can also use the findings of the current study to develop educational and therapeutic protocols and to examine their effectiveness in clinical trials.

## Conclusion

Following the insights from this study, it seems that evaluating self-efficacy and personality traits in methamphetamine users provides valuable information for clinical work, especially for substance abuse and its treatment. In line with the results of the present study, personality traits, and self-efficacy could predict mental health, sleep quality, and relapse, and there was a significant difference in terms of mental health, relapse, and sleep quality in the three clusters in this study.

In other words, these variables developed three distinct clusters, and the methamphetamine users in the first cluster with higher levels of neuroticism, sensation seeking, and aggressiveness, and low levels of extroversion, activity, and self-efficacy reported a low level of mental health and sleep quality but a higher relapse rate compared to the methamphetamine users in the second and third cluster.

Given the relatively high prevalence of methamphetamine use and its increasing trend in the community, the data in this study indicated that self-efficacy and personality traits could cluster methamphetamine users. This finding can have some implications for preventing and treating methamphetamine use. Accordingly, people with low self-efficacy or high neuroticism can be identified in educational centers such as schools and universities, and these variables can be adjusted with the help of treatment protocols because, in many cases, prevention works better than treatment. Furthermore, the mentioned variables should be seriously considered in the treatment centers, and effective solutions can be presented to the centers in charge of serving these people. As the findings indicated, relapse is associated with personality traits and self-efficacy; thus, reducing relapse is one of the main goals in treating methamphetamine users. However, in medical centers in Iran, more attention is paid to detoxification and drug treatments at the expense of underlying psychological variables. Accordingly, medical centers should address psychological factors as soon as clients are admitted to come up with more stable treatment outcomes.

## Data Availability

The datasets generated during and/ or analyzed during the current study are available from the Corresponding Author: Dr. Safora Salemi (email: safora.salemi@kums.ac.ir) on reasonable request.

## References

[1] Dean AC, Groman SM, Morales AM, London ED (2013). An evaluation of the evidence that methamphetamine abuse causes cognitive decline in humans. Neuropsychopharmacology.

[2] Gonzales R, Mooney L, Rawson RA (2010). The methamphetamine problem in the United States. Annu. Rev. Public Health.

[3] Jones CM, Compton WM, Mustaquim D (2020). Patterns and characteristics of methamphetamine use among adults—United States, 2015–2018. Morb. Mortal. Wkly. Rep..

[4] Alam-Mehrjerdi Z, Abdollahi M, Higgs P, Dolan K (2015). Drug use treatment and harm reduction programs in Iran: A unique model of health in the most populated Persian Gulf country. Asian J. Psychiatr..

[5] Rade CB, Desmarais SL, Van Dorn RA, Lutnick A, Kral AH, Lorvick J (2015). Mental health correlates of drug treatment among women who use methamphetamine. Am. J. Addict..

[6] Connery HS, McHugh RK, Reilly M, Shin S, Greenfield SF (2020). Substance use disorders in global mental health delivery: Epidemiology, treatment gap, and implementation of evidence-based treatments. Harvard Rev. Psychiatry.

[7] Baingana F, Al’absi M, Becker AE, Pringle B (2015). Global research challenges and opportunities for mental health and substance use disorders. Nature.

[8] Asselmann E, Wittchen HU, Lieb R, Höfler M, Beesdo-Baum K (2014). Associations of fearful spells and panic attacks with incident anxiety, depressive, and substance use disorders: A 10-year prospective-longitudinal community study of adolescents and young adults. J. Psychiatr. Res..

[9] Buckner JD, Schmidt NB, Lang AR, Small JW, Schlauch RC, Lewinsohn PM (2008). Specificity of social anxiety disorder as a risk factor for alcohol and cannabis dependence. J. Psychiatr. Res..

[10] Christie KA, Burke JD, Regier DA, Rae DS, Boyd JH, Locke BZ (1988). Epidemiologic evidence for early onset of mental disorders and higher risk of drug abuse in young adults. Am. J. Psychiatry.

[11] Sunderland M, Slade T, Krueger RF (2015). Examining the shared and unique relationships among substance use and mental disorders. Psychol. Med..

[12] Walsh Z, Gonzalez R, Crosby K, Thiessen MS, Carroll C, Bonn-Miller MO (2017). Medical cannabis and mental health: A guided systematic review. Clin. Psychol. Rev..

[13] van Emmerik-van OK, van de Glind G, van den Brink W, Smit F, Crunelle CL, Swets M (2012). Prevalence of attention-deficit hyperactivity disorder in substance use disorder patients: A meta-analysis and meta-regression analysis. Drug Alcohol Depend..

[14] Khantzian EJ (1997). The self-medication hypothesis of substance use disorders: A reconsideration and recent applications. Harvard Rev. Psychiatry.

[15] Quello SB, Brady KT, Sonne SC (2005). Mood disorders and substance use disorder: A complex comorbidity. Sci. Pract. Perspect..

[16] Lipinska G, Timol R, Thomas KGF (2015). The implications of sleep disruption for cognitive and affective processing in methamphetamine abuse. Med. Hypotheses.

[17] Sun-Suslow N, Saloner R, Serrano V, Umlauf A, Morgan EE, Ellis RJ (2020). Lifetime methamphetamine use disorder and reported sleep quality in adults living with HIV. AIDS Behav..

[18] London ED, Berman SM, Voytek B, Simon SL, Mandelkern MA, Monterosso J (2005). Cerebral metabolic dysfunction and impaired vigilance in recently abstinent methamphetamine abusers. Biol. Psychiatry.

[19] London ED, Simon SL, Berman SM, Mandelkern MA, Lichtman AM, Bramen J (2004). Mood disturbances and regional cerebral metabolic abnormalities in recently abstinent methamphetamine abusers. Arch. Gen. Psychiatry.

[20] Perez AY, Kirkpatrick MG, Gunderson EW, Marrone G, Silver R, Foltin RW (2008). Residual effects of intranasal methamphetamine on sleep, mood, and performance. Drug Alcohol Depend..

[21] Hser Y-I, Huang D, Chou C-P, Teruya C, Anglin MD (2003). Longitudinal patterns of treatment utilization and outcomes among methamphetamine abusers: A growth curve modeling approach. J. Drug Issues.

[22] Brecht ML, Herbeck D (2014). Time to relapse following treatment for methamphetamine use: A long-term perspective on patterns and predictors. Drug Alcohol Depend..

[23] Galanter M (2006). Innovations: Alcohol & drug abuse: Spirituality in alcoholics anonymous: A valuable adjunct to psychiatric services. Psychiatr. Serv..

[24] Kadden RM, Litt MD (2011). The role of self-efficacy in the treatment of substance use disorders. Addict. Behav..

[25] Bandura A (2004). Health promotion by social cognitive means. Health Educ. Behav..

[26] Dolan SL, Martin RA, Rohsenow DJ (2008). Self-efficacy for cocaine abstinence: Pretreatment correlates and relationship to outcomes. Addict. Behav..

[27] McKellar J, Ilgen M, Moos BS, Moos R (2008). Predictors of changes in alcohol-related self-efficacy over 16 years. J. Subst. Abuse Treat..

[28] Robinson SM, Walsh J (1994). Cognitive factors affecting abstinence among adolescent polysubstance abusers. Psychol. Rep..

[29] Epstein JA, Griffin KW, Botvin GJ (2000). A model of smoking among inner-city adolescents: The role of personal competence and perceived social benefits of smoking. Prev. Med..

[30] Tsai TY, Wang TY, Tseng HH, Chen KC, Chiu CJ, Chen PS (2022). Correlation between loneliness, personality traits, and treatment outcomes in patients with methamphetamine use disorder. Sci. Rep..

[31] Ma CH, Lin KF, Chen TT, Yu YF, Chien HF, Huang WL (2020). Specific personality traits and associated psychosocial distress among individuals with heroin or methamphetamine use disorder in Taiwan. J. Formosan Med. Assoc..

[32] Al-Turkait FA, Ohaeri JU (2016). Relationship of personality traits with anxiety, depressive and PTSD symptomatology and academic performance. Experience with an Arab college student sample. Lebanese Med. J..

[33] Magee CA, Heaven PC, Miller LM (2013). Personality change predicts self-reported mental and physical health. J. Person..

[34] Kroencke L, Kuper N, Bleidorn W, Denissen J (2020). How does substance use affect personality development? Disentangling between- and within-person effects. Soc. Psychol. Person. Sci..

[35] Tatari F, Farnia V, Lalehgni F, Moradi A, Radmehr F, Davarinejad O, Hookar S, Salemi S, Golmohamadi F, Alikhani M (2024). The efficacy of venlafaxine in the treatment of depression, withdrawal symptoms, and craving in individuals with methamphetamine dependence. J. Subst. Use.

[36] Dalmaijer ES, Nord CL, Astle DE (2022). Statistical power for cluster analysis. BMC Bioinform..

[37] Wong MC, Lau TC, Lee A (2012). The impact of leadership programme on self-esteem and self-efficacy in school: A randomized controlled trial. PLoS ONE.

[38] Rabbani Bavojdan M (2013). The relationship between self-efficacy beliefs and metacognition with coping strategies in male abusers. Appl. Psychol..

[39] Zuckerman M, Kuhlman DM, Joireman J, Teta P, Kraft M (1993). A comparison of three structural models for personality: The Big Three, the Big Five, and the Alternative Five. J. Person. Soc. Psychol..

[40] Rezaei M, Zakiei A, Reshadat S, Ghasemi SR (2017). The role of individual and personality factors in controlling risky behaviours related to AIDS: Proposing a causal model. Person. Mental Health.

[41] Sánchez-López Mdel P, Dresch V (2008). The 12-item general health questionnaire (GHQ-12): Reliability, external validity, and factor structure in the Spanish population. Psicothema.

[42] Ebadi M, Harirchi AM, Shariati M, Gannaroudi GR, Fateh A, Montazeri A (2002). Translation, determining the reliability and validity of the 12-question general health questionnaire (GHQ-12). Payesh.

[43] Zakiei A, Khazaie H, Moradi F, Komasi S (2020). Effects of combined profiles derived from sleep quality and disorders on non-suicidal self-injury (NSSI) behaviors. J. Turk. Sleep Med..

[44] Yamashita A, Yoshioka S-I, Yajima Y (2021). Resilience and related factors as predictors of relapse risk in patients with substance use disorder: A cross-sectional study. Subst. Abuse Treat. Prev. Policy.

[45] Yamini M, Khorsandi Shamir Y (2024). Psychometric properties of the stimulant relapse risk scale (SRRS). Recent Innov. Psychol..

[46] Yıldırım M, Güler A (2022). COVID-19 severity, self-efficacy, knowledge, preventive behaviors, and mental health in Turkey. Death Stud..

[47] Gloudemans HA, Schalk RM, Reynaert W (2013). The relationship between critical thinking skills and self-efficacy beliefs in mental health nurses. Nurse Educ. Today.

[48] Grevenstein D, Bluemke M, Kroeninger-Jungaberle H (2016). Incremental validity of sense of coherence, neuroticism, extraversion, and general self-efficacy: Longitudinal prediction of substance use frequency and mental health. Health Qual. Life Outcomes.

[49] Schlarb AA, Kulessa D, Gulewitsch MD (2012). Sleep characteristics, sleep problems, and associations of self-efficacy among German university students. Nat. Sci. Sleep.

[50] Byun E, McCurry SM, Opp M, Liu D, Becker KJ, Thompson HJ (2020). Self-efficacy is associated with better sleep quality and sleep efficiency in adults with subarachnoid haemorrhage. J. Clin. Neurosci..

[51] Ghodrati Mirkouhi M, Hadadi S, Akbari-Kamrani M (2018). Correlation between self-efficacy with sleep quality and sexual function index in patients with type 2 diabetes. Qom Univ. Med. Sci. J..

[52] Miró E, Martínez MP, Sánchez AI, Prados G, Medina A (2011). When is pain related to emotional distress and daily functioning in fibromyalgia syndrome? The mediating roles of self-efficacy and sleep quality. Br. J. Health Psychol..

[53] Tighe CA, Youk A, Ibrahim SA, Weiner DK, Vina ER, Kwoh CK (2020). Pain catastrophizing and arthritis self-efficacy as mediators of sleep disturbance and osteoarthritis symptom severity. Pain Med. (Malden, Mass.).

[54] Simonetti V, Durante A, Ambrosca R, Arcadi P, Graziano G, Pucciarelli G (2021). Anxiety, sleep disorders and self-efficacy among nurses during COVID-19 pandemic: A large cross-sectional study. J. Clin. Nurs..

[55] Cong L, Ju Y, Gui L, Zhang B, Ding F, Zou C (2021). The mediating role of self-efficacy in sleep disorder and depressive symptoms among Chinese caregivers of stroke inpatients: A structural equation modeling analysis. Neuropsychiatr. Dis. Treat..

[56] Zhang Y, Feng B, Geng W, Owens L, Xi J (2016). "Overconfidence" versus "helplessness": A qualitative study on abstinence self-efficacy of substance users in a male compulsory drug detention center in China. Subst. Abuse Treat. Prev. Policy.

[57] Schuck K, Otten R, Kleinjan M, Bricker JB, Engels RC (2014). Self-efficacy and acceptance of cravings to smoke underlie the effectiveness of quitline counseling for smoking cessation. Drug Alcohol Depend..

[58] Petraitis J, Flay BR, Miller TQ (1995). Reviewing theories of adolescent substance use: Organizing pieces in the puzzle. Psychol. Bull..

[59] Hiemstra M, Otten R, Engels RC (2012). Smoking onset and the time-varying effects of self-efficacy, environmental smoking, and smoking-specific parenting by using discrete-time survival analysis. J. Behav. Med..

[60] Li M, Ahmed MZ, Hiramoni FA, Zhou A, Ahmed O, Griffiths MD (2021). Mental health and personality traits during COVID-19 in China: A latent profile analysis. Int. J. Environ. Res. Public Health.

[61] Shokrkon A, Nicoladis E (2021). How personality traits of neuroticism and extroversion predict the effects of COVID-19 on the mental health of Canadians. PLoS ONE.

[62] Kotov R, Gamez W, Schmidt F, Watson D (2010). Linking, "big" personality traits to anxiety, depressive, and substance use disorders: A meta-analysis. Psychol. Bull..

[63] Jarrin DC, Chen IY, Ivers H, Morin CM (2014). The role of vulnerability in stress-related insomnia, social support, and coping styles on incidence and persistence of insomnia. J. Sleep Res..

[64] Van Reeth O, Weibel L, Spiegel K, Leproult R, Dugovic C, Maccari S (2000). Physiology of sleep (review)—Interactions between stress and sleep: From basic research to clinical situations. Sleep Med. Rev..

[65] Cellini N, Duggan KA, Sarlo M (2017). Perceived sleep quality: The interplay of neuroticism, affect, and hyperarousal. Sleep Health.

[66] Farhadi Afshar P, Banisi P (2022). Prediction of sleep quality based on aggression and anxiety in students of West Tehran Branch of Islamic Azad University. New Approach Educ. Sci..

[67] Azimi Z, Haghayegh SA, Norouzi M (2019). The moderating role of sleep quality in the relationship between state-trait anxiety with the severity of symptoms in patients with a migraine headache diagnosis. Q. J. Health Psychol..

[68] Schellekens AF, de Jong CA, Buitelaar JK, Verkes RJ (2015). Co-morbid anxiety disorders predict early relapse after inpatient alcohol treatment. Eur. Psychiatry J. Assoc. Eur. Psychiatr..

[69] Fisher LA, Elias JW, Ritz K (1998). Predicting relapse to substance abuse as a function of personality dimensions. Alcohol. Clin. Exp. Res..

[70] Hojjat SK, Golmakani E, Bayazi MH, Mortazavi R, Norozi Khalili M, Akaberi A (2015). Personality traits and identity styles in methamphetamine-dependent women: A comparative study. Glob. J. Health Sci..

[71] Baron-Oladi S, Navidian A, Kaveh-Farsani Z (2013). The study of the relationship between addiction potentiality and personality traits, conformity and gender among pre-university students. J. Shahrekord Univ. Med. Sci..

[72] Mahmoodi T, Bassaknezad S, Mehrabizadeh M (2020). Role of anger rumination and cognitive emotion regulation strategies in prediction of sleep quality in female students. Knowl. Res. Appl. Psychol..

[73] Taghvaee L, Mazandarani AA (2022). Poor sleep is associated with sensation-seeking and risk behavior in college students. Sleep Sci..

[74] Rusnac N, Spitzenstetter F, Tassi P (2019). Chronic sleep loss and risk-taking behavior: Does the origin of sleep loss matter?. Behav. Sleep Med..

[75] Tonetti L, Adan A, Caci H, De Pascalis V, Fabbri M, Natale V (2010). Morningness–eveningness preference and sensation seeking. Eur. Psychiatry J. Assoc. Eur. Psychiatr..

[76] Hampson SE, Andrews JA, Barckley M (2008). Childhood predictors of adolescent marijuana use: Early sensation-seeking, deviant peer affiliation, and social images. Addict. Behav..

[77] Martins SS, Storr CL, Alexandre PK, Chilcoat HD (2008). Adolescent ecstasy and other substance use in the National Survey of Parents and Youth: The role of sensation-seeking, parental monitoring, and peer's substance use. Addict. Behav..

[78] Dom G, Hulstijn W, Sabbe B (2006). Differences in impulsivity and sensation seeking between early- and late-onset alcoholics. Addict. Behav..

[79] Rasoli Z, Eslami R, Khademi A (2015). Evaluation of the relationship between emotional intelligence and mental health and anger among the Air force personnel. EBNESINA.

[80] Kayser MS, Mainwaring B, Yue Z, Sehgal A (2015). Sleep deprivation suppresses aggression in Drosophila. eLife.

[81] McCormick RA, Smith M (1995). Aggression and aggressiveness in substance abusers: The relationship to abuse patterns, coping style, and relapse triggers. Addict. Behav..

[82] Plüddemann A, Flisher AJ, McKetin R, Parry C, Lombard C (2010). Methamphetamine use, aggressive behavior and other mental health issues among high-school students in Cape Town, South Africa. Drug Alcohol Depend..

[83] Loree AM, Lundahl LH, Ledgerwood DM (2015). Impulsivity as a predictor of treatment outcome in substance use disorders: Review and synthesis. Drug Alcohol Rev..

[84] Umegaki Y, Higuchi A (2022). Personality traits and mental health of social networking service users: A cross-sectional exploratory study among Japanese undergraduates. Comput. Hum. Behav. Rep..

[85] Klinger-König J, Hertel J, Terock J, Völzke H, Van der Auwera S, Grabe HJ (2018). Predicting physical and mental health symptoms: Additive and interactive effects of difficulty identifying feelings, neuroticism, and extraversion. J. Psychosom. Res..

[86] Kardum I, Krapić N (2001). Personality traits, stressful life events, and coping styles in early adolescence. Pers. Individ. Differ..

[87] Haren EG, Mitchell CW (2003). Relationships between the five-factor personality model and coping styles. Psychol. Educ..

[88] Stephan Y, Sutin AR, Bayard S, Križan Z, Terracciano A (2018). Personality and sleep quality: Evidence from four prospective studies. Health Psychol..

[89] Hintsanen M, Puttonen S, Smith K, Törnroos M, Jokela M, Pulkki-Råback L (2014). Five-factor personality traits and sleep: Evidence from two population-based cohort studies. Health Psychol..

[90] Gray EK, Watson D (2002). General and specific traits of personality and their relation to sleep and academic performance. J. Person..

[91] Strickhouser JE, Zell E, Krizan Z (2017). Does personality predict health and well-being? A meta-synthesis. Health Psychol..

[92] Munafò MR, Black S (2007). Personality and smoking status: A longitudinal analysis. Nicotine Tob. Res..

[93] Enns MW, Cox BJ (1997). Personality dimensions and depression: Review and commentary. Can. J. Psychiatry.

